# Asymmetric Dimethylarginine as a Surrogate Marker of Endothelial Dysfunction and Cardiovascular Risk in Patients with Systemic Rheumatic Diseases

**DOI:** 10.3390/ijms131012315

**Published:** 2012-09-26

**Authors:** Theodoros Dimitroulas, Aamer Sandoo, George D. Kitas

**Affiliations:** 1 Department of Rheumatology, Dudley Group NHS Foundation Trust, Russells Hall Hospital, Dudley, West Midlands DY1 2HQ, UK; E-Mails: aamer.sandoo@dgoh.nhs.uk (A.S.); kitas@dgh.nhs.uk (G.D.K.); 2 Arthritis Research UK Epidemiology Unit, University of Manchester, Manchester M15 6SZ, UK

**Keywords:** ADMA, rheumatic diseases, rheumatoid arthritis, atherosclerosis, cardiovascular disease

## Abstract

The last few decades have witnessed an increased life expectancy of patients suffering with systemic rheumatic diseases, mainly due to improved management, advanced therapies and preventative measures. However, autoimmune disorders are associated with significantly enhanced cardiovascular morbidity and mortality not fully explained by traditional cardiovascular disease (CVD) risk factors. It has been suggested that interactions between high-grade systemic inflammation and the vasculature lead to endothelial dysfunction and atherosclerosis, which may account for the excess risk for CVD events in this population. Diminished nitric oxide synthesis—due to down regulation of endothelial nitric oxide synthase—appears to play a prominent role in the imbalance between vasoactive factors, the consequent impairment of the endothelial hemostasis and the early development of atherosclerosis. Asymmetric dimethylarginine (ADMA) is one of the most potent endogenous inhibitors of the three isoforms of nitric oxide synthase and it is a newly discovered risk factor in the setting of diseases associated with endothelial dysfunction and adverse cardiovascular events. In the context of systemic inflammatory disorders there is increasing evidence that ADMA contributes to the vascular changes and to endothelial cell abnormalities, as several studies have revealed derangement of nitric oxide/ADMA pathway in different disease subsets. In this article we discuss the role of endothelial dysfunction in patients with rheumatic diseases, with a specific focus on the nitric oxide/ADMA system and we provide an overview on the literature pertaining to ADMA as a surrogate marker of subclinical vascular disease.

## 1. Introduction

Over the last years the importance of vascular disease in terms of cardiovascular morbidity and global mortality in patients with systemic rheumatic diseases has been extensively investigated and recognized. As demonstrated by observational studies [1,2] and a recent meta-analysis [3], cardiovascular disease (CVD) is the biggest contributor to the increased mortality observed in Rheumatoid Arthritis (RA), which is in common with other connective tissue diseases [4]. Traditional CVD risk factors [5–7] along with emerging CVD risk factors such as inflammatory mediators, chronic corticosteroid consumption, spasm of coronary arteries, derangement of fibrinolysis and platelet activation have been reported to be more prevalent in patients with systemic inflammatory diseases [8–10].

For many decades the vascular involvement in autoimmune disorders was synonymous with the presence of inflammatory infiltration in the vessel walls—the vasculitic lesions. Recent insights elucidating inflammation and autoimmunity as important contributing factors to the pathogenetic mechanisms of atherosclerosis, have stimulated renewed interest in the vascular damage and the associated CVD risk in patients with rheumatic diseases. Inflammation in this population has a systemic nature and it has been suggested that inflammatory molecules and cytokines originating from the inflamed synovium or the liver may have systemic vascular consequences resulting in endothelial dysfunction which is a pivotal early step in atherogenesis [11]. Given that pro-atherogenic lipid abnormalities [12] and elevated serum levels of *C*-reactive protein (CRP) [13] have been described before the onset of joint symptoms in RA, it has been suggested that the pathophysiological process may actually precede the clinical appearance of the disease. Therefore, it is not surprising that endothelial dysfunction has been observed in RA [14] and psoriatic arthritis [15] without any traditional CVD risk factors or history of CVD disease. Endothelial cell abnormalities are also common in asymptomatic individuals with CVD risk factors [16] and they convey prognostic significance regarding the occurrence of CVD events [17]. Thus, the recognition of early signs of impaired endothelial function could contribute to the introduction of protection measures at the time that they are more likely to be effective and the reduction of CVD co-morbidity, particularly in patients with arthritides and connective tissue disease.

A growing amount of literature reports the use of functional and morphological markers for the assessment of endothelial function in systemic inflammatory diseases. For example increased intima-media thickness, arterial stiffness as well as *in vivo* assessment of the micro and microvasculature have been used as surrogate markers in the study of accelerated atherosclerosis in various rheumatic disease subsets. Most of them have shown advanced functional and morphological abnormalities in RA [18–21], other inflammatory arthropathies [22,23], systemic lupus erythematosus [24–26] and systemic sclerosis [27]. Endothelium derived biomarkers such as adhesion molecules (e.g., *E*-selectin), Von Willebrand factor, thrombomodulin as well as other serological indicators of CVD risk such as prothrombotic factors, uric acid and osteoprotegerin have also been used as markers of endothelial function or activation or both [28–32]. However most of them are subject to multiple confounding variables and have pleiotropic biological effects which may vary over time and with different types or stages of disease in patients with autoimmune disorders. Additionally their utility has not been validated in well-controlled studies in large cohorts of patients, as the majority of studies performed to-date are cross-sectional or small longitudinal observation cohorts. As a result their potential clinical implication remains unclear.

Assymetric dimethylarginine (ADMA) is an endogenous guanido-substituted analogue of L-arginine and decreases the bioavailability of nitric oxide (NO) by inhibiting the three isoforms of nitric oxide synthase (NOS). Several lines of evidence suggest that elevated ADMA levels mediate endothelial dysfunction in various conditions associated with atherosclerosis such as lipid disorders, hypertension, peripheral artery disease and chronic kidney disease [33]. In the field of rheumatology, there is growing interest in the role of ADMA as a biochemical marker of endothelial impairment and subclinical atherosclerosis and some studies have addressed its utility and clinical significance in different vascular beds and disease states. The aim of the present article is to provide an overview of the current data supporting the involvement of ADMA in accelerated atherosclerosis and CVD complications associated with systemic rheumatic diseases. First the role of NO/ADMA pathway in the vascular and endothelium hemostasis is discussed.

## 2. The Role of NO in Health and in Inflammation: The Beauty and the Beast?

The endothelium is a flat monolayer of cells between the vascular lumina and the blood and it is present throughout the body. It forms an important part of the vasculature as it represents the principal regulator of vascular function through the maintenance of vascular tone and through the release of a variety of vasoactive factors, hormones and neurotransmitters which affect vascular permeability, migration of leucocytes and inflammation. The balanced production of the vasoactive substances is atheroprotective via the regulation of fibrinolysis and coagulation, whereas a damaged endothelium causes disrupted production with the ensuing imbalance resulting in endothelial dysfunction, an early marker of atherosclerosis [34].

One of the most important endothelium-derived vasodilatory mediators is the free radical NO which is synthesized through the conversion of amino acid L-arginine to NO under the governance of NOS [35]. Three isoforms of NOS have been described: neuronal NOS which produces NO in nervous tissue to act as neuronal messenger, macrophage or inducible NOS which is only released in an oxidated environment after activation of macrophages, and endothelial NOS which generates NO in blood vessels [36]. Once produced, NO diffuses across the endothelial cell and acts on smooth muscle cells to induce vasodilation by increasing production of the second messenger cyclic guanosine monophosphate via activation of soluble guanylate cyclase. Constitutive production of NO is important for the regulation of blood flow, the maintenance of vasorelaxation and the prevention of of smooth muscle cells proliferation. Apart from the vasodilatory properties, NO also inhibits platelet aggregation and leucocyte adhesion to the vessel wall protecting the vascular endothelium from oxidative injury [37]. Although other relaxing or constricting factors such as prostacyclin, endothelin-1 and endothelium-derived hyperpolarizing factor are strongly associated with vascular homeostasis, their contribution appears to be greater in conditions of deficient NO release [38].

Pro-inflammatory mediators and cytokines may exert deleterious effects on the endothelial cells leading to diminished synthesis of NO. Endothelial dysfunction defined as loss of endothelial adaption to vasoactive stimuli, is characterized by the loss of protective endothelial characteristics [39]. The down-regulation of endothelial NOS and the consequent reduction in NO bioactivity, accompanied by the activation of endothelial and inflammatory cells, results in dysregulation of vascular tone, progressive disorganization of the vascular architecture and increased expression of adhesion molecules; functional and structural changes that promote thrombosis and atherosclerosis [40].

It is worth noting that NO has a dual role in physiological and pathological conditions, one protective and one injurious, mainly depending on the concentration and the local environment. CRP inhibits the activity of the endothelial NOS via uncoupling of the enzyme both *in vivo* and *in vitro* [41]. In systemic inflammatory conditions, NOS changes from the endothelial form to the inducible form, and NO produced by the modulation of inducible NOS react with free radicals released by inflammatory cells to form peroxynitrate which then mediates cellular and tissue injury. Although it has been suggested that the decreased phosphorylation of endothelial NOS reduces bioavailability of NO particularly in the context of RA [42], overexpression of inducible NOS and subsequent upregualtion of NO production has been shown to induce oxidative vascular damage and endothelial cell apoptosis in hypoxic conditions [43]. These biphasic effects of NO on vascular endothelium including its transformation from a protector to an enhancer of vascular injury are typical in systemic sclerosis (SSc) [44].

## 3. ADMA as a Mediator of Cardiovascular Disease

### 3.1. Biology

**Figure 1:**
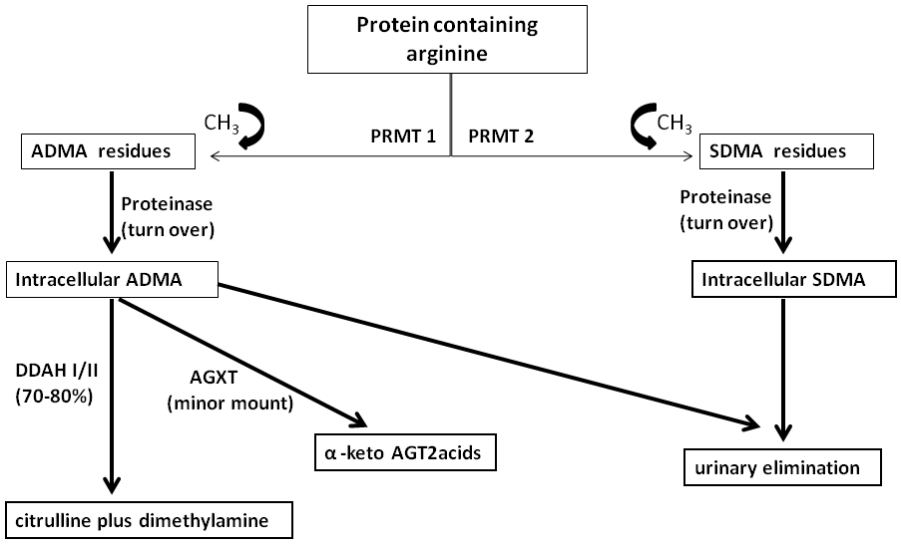
Synthesis and metabolism of dimethylarginines. ADMA: asymmetric dimethylarginine, SDMA: symmetric dimethylarginine, PMRT: protein-arginine ethyl transferase, DDAH: dimethylarginine dimethylamonohydrolase, AGXT: alanine-glyoxylate aminotransferase.

ADMA is a naturally occurring component of human blood plasma. It is produced by methylation of arginine residues, a common mechanism of post-translational modification of the tertiary structure and the function of proteins. The methylation is carried out by a group of enzymes referred to as protein-arginine methyl transferase’s (PRMT’s). The complex name of these enzymes suggests their molecular function: they transfer one or more methyl groups from the methyl group donor *S*-adenosylmethionine to L-arginine residues within proteins or polypeptides [45]. After proteolysis of methylated nuclear proteins, free dimethylarginines are released by the cells. PRMT I catalyzes asymmetrical dimethylation and monomethylation of arginine residues and produces ADMA, whereas type II catalyzes symmetrical dimethylation and monomethylasion and forms symmetric dimethylarginine—the biologically inactive stereoisomer of ADMA [46]. Free circulating ADMA is then released after degradation of such methylated protein residues. It has been reported that humans generate approximately 300 mol of ADMA per day in normal conditions [47]. About 70%–80% of ADMA is intracellularly hydrolyzed into citrulline and dimethylamine by the dimethylarginine dimethylamonohydrolase (DDAH) [48]. In addition, ADMA is also cleaved to α-keto valeric acid by alanine-glyoxylate aminotransferase [49], although the role of this pathway on total ADMA metabolism remains unclear (Figure 1).

### 3.2. ADMA and the Regulation of Vascular Tone

DDAH exists in at least two isoforms (DDAH-1, DDAH-2) with distinct tissue-relevant distributions but seemingly similar activities [50]. Recent insights have demonstrated that diminished DDAH activity is evident in a variety of conditions associated with endothelial dysfunction and it is considered as one of the potent mechanisms responsible for increased methylarginines and subsequent ADMA mediated eNOS impairment. The overall production of ADMA by endothelial cells is a balance between rates of arginine methylation, rates of post-translational protein degradation, rates of hydrolyitc cleavage by DDAH and active ADMA migration from the cell. Although the degree of contribution of each pathway in not known, it seems that the bioactivity of DDAH is the major determinant of intracellular ADMA concentration [51]. Animal models using *DDAH* gene silencing techniques and DDAH transgenic mice have provided evidence for the role of this enzyme in regulating vascular tone. Both deleting the DDAH-1 gene in mice and inhibiting its activity through DDAH-specific inhibitors resulted in functional endothelial changes, increased systemic vascular resistance and abnormal systemic blood pressure [52]. Moreover, increased levels and reduced catabolism of ADMA due to suppression of endothelium DDAH expression was found in both human lung tissue of pulmonary hypertension patients and the tissue of monocrotoline induced pulmonary hypertension in rats [53]. Last but not least enhancement of DDAH-1 expression increases basal levels of vascular NO and protects against ADMA-induced endothelial dysfunction in the cerebral circulation [54]. Recently it has been proposed that DDAH may also regulate vascular tone and haemostasis through mechanisms independent of ADMA mediated NOS inhibition [55].

### 3.3. ADMA and CVD

Data of clinical and experimental studies suggest that accumulation of ADMA contributes to reduced generation of NO in different disease subsets associated with endothelial dysfunction. Derangement of NO/ADMA pathway has been described in a wide range of CVD diseases as well as in patient populations with almost any traditional and emerging CVD risk factor, suggesting that ADMA is an early marker of atherosclerotic vascular disease [56]. For example, prospective investigations of ADMA have highlighted its significance as a predictor of major CVD events and deaths in patients with established coronary artery disease [57], diabetes mellitus [58] and advanced kidney disease [59]. Besides the predictive value regarding future adverse CVD outcome, quantification of ADMA with *N*-terminal pro brain natriuretic peptide—a well established and widely utilised in daily clinical setting biochemical tool in heart failure—has been additionally found to improve the risk stratification in this population [60]. Recently Tutarel *et al*. [61] found ADMA to be more sensitive than *N*-terminal pro brain natriuretic peptide for the assessment of functional status in patients with congenital heart disease, reinforcing the hypothesis that prolonged NOS downregulation may be involved in the hemodynamic changes that occur in chronic heart disease. With regards to high-risk populations, elevated ADMA levels are associated with increased mortality among haemodialysis patients [62] and patients recovering from acute myocardial infarction [63], and they have been reported as a causative factor in the development of multiple organ failure in critically ill patients admitted in the intensive care unit [64].

On the other hand the relevance of ADMA as a prospective risk factor in the general population has not been extensively studied thus far, as it requires large cohorts and very long follow-up periods. In a community-based epidemiological study—Framingham Offspring cohort—comprising of 3320 patients, ADMA levels were significantly related to all-cause mortality [65], however, the association of ADMA with the incidence of CVD events did not remain significant after 24 years. Positive correlation between ADMA and CVD related morbidity and mortality have also been reported in a few small cohorts but their results are difficult to extrapolate to the broader, as their population were not representative, consisted only of women [66] and non-smoking men [67]. Further large epidemiological studies and meta-analyses are advocated to elucidate whether ADMA represents a risk factor of CVD disease and events in the general population.

## 4. Cardiovascular Involvement in Rheumatic Diseases

Autoimmunity and inflammation forms the basis of the rheumatological diseases and contribute to the diversity of their clinical manifestations. In the context of CVD, the intensity of systemic inflammation underlies structural and functional changes of the heart, coronary, pulmonary and peripheral vasculature in inflammatory arthropathies [68] and connective tissue disease [69]. Although cardiac disease in autoimmune disorders can clinically present in many guises (such as heart failure, coronary artery disease, myocarditis, pulmonary hypertension (PH), pericarditis, valvular disease, *etc*.), it is those with an underlying ischaemic pathology that present more frequently and pose the greatest mortality risk, particularly in terms of RA [8,70].

In patients with immune diseases, the atherosclerotic lesions have a silent presentation and follow a more rapid evolution than in the general population and the terms “accelerate” and “premature” atherosclerosis have been proposed, to highlight the ‘magnitude of this process [71–73]. RA is now considered as a coronary heart disease equivalent [74] given that the excessive CVD risk profile is comparable to the risk conferred by type 2 diabetes in the general population [75]. The precise mechanisms responsible for this phenomenon are not clear but striking similarities between the inflammatory pathways in atherosclerosis and RA have suggested the fundamental role of high-grade systemic inflammation. However a recent systematic review [76] did not show a robust relationship between inflammation and vascular abnormalities in RA, indicating that the inflammatory process is not the sole factor accelerating rheumatoid vascular pathology. Advanced atherosclerotic plaque instability is considered one more contributing factor to the higher fatality rates and worse outcome from acute coronary syndromes observed in RA patients [77]. The modern view suggests that traditional CVD risk factors—which are operational in patients with autoimmune diseases—inflammation, production of autoantibodies, genetic predisposition [78,79] and their complex interrelations contribute to the enhanced CVD co-morbidity in this population [80].

Apart from atherosclerosis-related ischaemic vascular disease, systemic heart involvement and pulmonary vasculopathy represent important aspects of CVD present in a wide spectrum of connective tissue disorders such as SSc, mixed connective tissue disease and systemic lupus erythematosus. In terms of SSc, myocardial fibrosis and microvasular ischaemia due to direct vascular, fibrotic and inflammatory changes of the disease itself lead to systolic and diastolic abnormalities, conduction system and rhythm disturbances, and in advanced stages, to congestive heart failure [81]. SSc-related pulmonary arterial hypertension is a widely recognized complication which occurs as a result of direct proliferative pulmonary vascular remodeling. Both scleroderma cardiac disease and pulmonary hypertension exhibit few symptoms and outward clinical signs but when clinically established, they convey poor prognosis, representing the leading cause of death in SSc [82]. The early detection of cardiopulmonary involvement by simple, non-invasive methods is clearly desirable and recently the introduction of biochemical markers [83,84] and more sophisticated imaging modalities [85,86] have improved our ability to detect subclinical disease and accordingly, refer high-risk patients for further investigations.

In that respect ADMA has been investigated as surrogate marker of endothelial dysfunction, atherosclerosis, heart and/or pulmonary disease in the setting of different rheumatic disease subsets.

## 5. ADMA as Biochemical Marker of CVD in Rheumatic Diseases

### 5.1. Atherosclerotic Disease

Over the last years several studies have assessed the role of ADMA in the pathogenesis of atherosclerosis in systemic inflammatory disease. They have demonstrated higher ADMA levels in patients with early RA [87–89], psoriatic arthritis [90] and ankylosing spondylitis [91,92], even in the absence of clinical signs and CVD risk factors. Accumulation of ADMA is a result of increased production and/or reduced degradation. Enhanced TNF-alpha expression [93] and hypoxia within the inflamed synovium [94] have been reported to downregulate DDAH, whilst oxidative stress associated with RA increases the formation of ADMA via augmented expression of PRMTs [95]. The proliferation and potentiated apoptosis of vascular endothelial cells in the inflamed synovial tissue and the consequent release of ADMA during protein degradation may represent another possible mechanism of increased ADMA levels [96].

ADMA has been associated with various morphological and functional parameters of subclinical vascular disease in patients with autoimmune disorders indicating its role as a marker of endothelial dysfunction and premature atherosclerosis. Significant correlations have been established between ADMA and carotid artery intima-media thickness [87] as well as coronary flow reserve in patients with early RA [88] and PsA [90]. In contrast no significant relationship between ADMA and assessments of *in vivo* endothelium-dependent and -independent microvascular and macrovascular function was established in 67 RA patients with moderate disease activity [89]. Kiani *et al*. [97] reported a significant correlation between coronary calcium—a marker of early atherosclerosis—and ADMA in systemic lupus erythematosus patients. In the same disease setting, ADMA has also been associated with arterial stiffness [98] and CVD events including coronary artery disease, ischemic cerebrovascular events and peripheral artery disease [99].

The mechanisms through which ADMA promotes endothelial dysfunction are not fully understood. Defective vascular repair has been proposed as a process for premature atherosclerosis in rheumatic diseases and decreased numbers of circulating endothelial-progenitor cells have been described in RA [100] and systemic lupus erythematosus [101]. Although enhanced migration of endothelial cell precursors into the inflamed synovium may account for the depletion of their circulating number in RA, it is known that the mobilization of progenitor cells from bone marrow in response to various stimuli is dependent on eNOS activity [102]. Surdacki *et al.* [87] showed an inverse correlation between ADMA and circulating endothelial progenitor cells in RA patients, suggesting that ADMA mediated deficit of endothelium-derived NO, may contribute to the diminished capacity of endothelial repair and vascular remodelling, translated into acceleration of atherogenesis and plaque destabilization and consequently into augmented CVD risk.

As mentioned above the inflammatory component is considered an important factor of increased CVD risk in patients with systemic rheumatic diseases and combined with other conventional and disease-related factors leads to diverse clinical presentations. The association between endothelial dysfunction and inflammation particularly in high-grade systemic inflammatory disorders is questionable. ADMA has been associated with inflammatory indicators only in some studies in patients with arthritides [92,103]. Additionally, clinical remission following treatment with anti-rheumatic medications is not accompanied by changes in ADMA in patients with RA in two small longitudinal cohorts [104,105]. Insulin resistance, but not inflammatory markers, has been reported as an independent predictor of elevated ADMA levels in RA [106], and this is concurrent with preliminary results in patients with hypertension [107].

Besides systemic inflammation, another hallmark of autoimmune diseases is immune dysregulation, manifested by the presence of autoantibodies, which may also be important in mediating CVD risk. Rheumatoid factor and antinuclear antibodies positive subjects carry increased risk of CVD events even after adjusting for the presence of rheumatic disease [108]. Also anti-CCP antibodies are connected with impaired endothelial function and myocardial involvement in RA [109,110]. Thus, it is not surprising that ADMA is associated with ds-DNA anti-SM, anti-RNP and low complement in patients with systemic lupus erythematosus [97,99]. Also accumulation of ADMA is related to anti-CCP antibodies in patients with early RA [111], providing a link between endothelial dysfunction and autoimmune dysregulation, two important indices of excess CVD risk in systemic rheumatic diseases.

**Figure 2:**
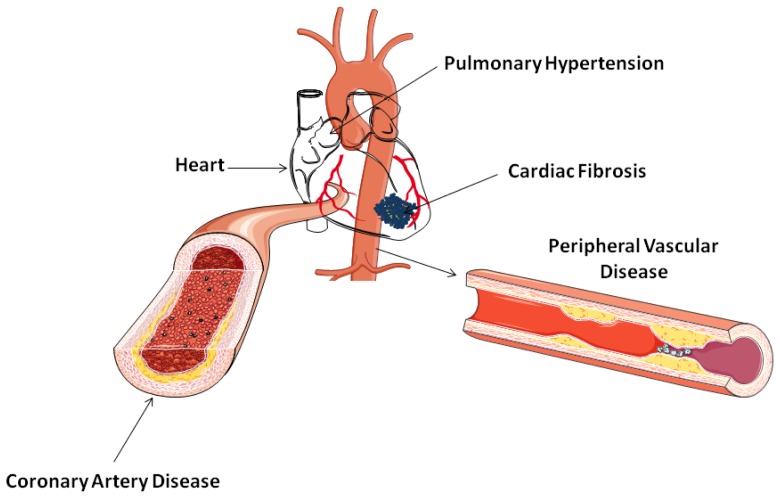
Cardiovascular complications in rheumatic diseases have been linked with ADMA. Enhanced morbidity and mortality due to increased incidence of atherosclerosis and ischemic heart disease contribute to the reduced life expectancy reported in patients with inflammatory arthropathies and connective tissue diseases. Widespread endothelial dysfunction and disturbance of coronary and pulmonary microcirculation results in myocardial fibrosis and pulmonary hypertension in patients with scleroderma and related syndromes. Peripheral vascular involvement with digital ulceration is a common clinical feature of systemic vasculities and scleroderma.

The studies discussed above underlie the complexion of the interplays between vascular pathology, modifiable (inflammation, insulin sensitivity, lipids *etc*.) and non-modifiable (age, sex, autoantibodies, genes *etc*.) risk factors, suggesting that future work should focus on delineating the exact mechanisms involved, and evaluate the role of ADMA as a risk assessment tool which encompasses the different pathophysiological pathways contributing to the increased CVD morbidity in this population (Figure 2).

### 5.2. Cardiac Involvement and Pulmonary Hypertension

Endothelial dysfunction due to reduced bioavailability of NO is thought to play an important role in the development of pulmonary hypertension. Abnormal NO metabolism accompanied by administration of ADMA has been described in patients with pulmonary hypertension associated with advanced systolic heart failure [112] and Down syndrome [113]. Elevated ADMA concentrations are also associated with hemodynamic parameters and prognosis in idiopathic [114] and chronic thromboembolic pulmonary hypertension [115]. With regards to SSc, it has been reported that ADMA is increased in patients with echocardiographically defined pulmonary hypertension [116] and it is correlated with systolic pulmonary artery pressure and functional capacity [117]. Given that exhaled NO is diminished in patients with SSc and pulmonary hypertension [118], it is tempting to speculate that the inhibition of endothelial NOS in the lungs by ADMA, may contribute to pulmonary vasoconstriction and to the proliferation and hypertrophy of the tunica media, typically observed in SSc-related pulmonary vascular disease.

The multi-facet involvement of NO/ADMA pathway in cardiac pathophysiology is well-recognized. Positive correlations between ADMA and echocardiographic indices of left ventricle diastolic dysfunction have been established in patients with SSc and subclinical heart disease [119]. Such associations have also been found in patients with chronic heart failure [120]. It has been reported that long-term inhibition of NO stimulates the synthesis of transforming growth factor β in rats leading to cardiac fibrosis [121]. The fact that transforming growth factor β is considered as one of the most important cytokines involved in the pathogenesis of SSc, may indicate a potent etiopathogenetic role of ADMA in SSc heart disease and myocardial dysfunction. Additional investigations are warranted to confirm these findings and clarify the role of ADMA as an alternative tool to monitor disease severity and predict clinical deterioration in patients with rheumatic diseases and cardiopulmonary complications.

### 5.3. Peripheral Vascular Disease

Generalised vasculopathy, endothelial injury and disturbed angiogenesis are amongst the primary events in the pathogenesis of SSc. The involvement of the microvasculature is clinically manifested with Raynaud’s, a transient vasospastic phenomenon characterized by cold-induced digital ischemic attacks. Elevated ADMA concentrations in patients with secondary Raynaud’s [122] and diffuse cutaneous disease [123] are considered as the reflection of endothelial insult through both reduced and enhanced NO production [44]. Besides the inhibition of endothelial NOS, deranged NO regulation due to overexpression of inducible NOS, occurs in patients with SSc [124] and possibly contributes to accumulation of ADMA by eliminating DDAH activity [125]. ADMA levels in SSc have also been correlated with markers of matrix remodelling [126] but not with biochemical indicators of abnormal angiogenesis [127].

Critical lower and upper limb ischemia is not uncommon in patients with inflammatory vascular disease, mainly in the context of SSc and vasculitis. In cases refractory to oral vasodilators and/or anti-rheumatic treatment, iloprost, a synthetic analogue of prostacyclin, is used with good clinical outcomes. On the top of the vasodilatory effects, iloprost may also convey immunomodulating properties as it significantly decreases the levels of circulating endothelial adhesion molecules in rheumatoid vasculitis [128]. Intravenous infusion of iloprost led to reduction of ADMA in a small longitudinal study including patients with peripheral arterial occlusive disease, and this change was also associated with clinical improvement [129]. Whether ADMA is eligible as a biomarker for the treatment guidance and the evaluation of the responsiveness of vasodilators in severe inflammatory vascular disease, remains to be determined in future studies.

Table 1 summarizes the studies assessing the role of ADMA in various rheumatic diseases.

## 6. Conclusions and Future Implications

In patients with systemic autoimmune diseases, the prevention of CVD risk is now regarded as part of the global management along with controlling of disease activity and inflammation. Endothelial dysfunction and accelerated atherosclerosis are present in the early years of the disease before conventional CVD risk factors or cardiac disease become apparent. The widening of the mortality gap between RA patients and the general population [130], calls for early identification of patients at higher risk in order to improve outcomes and introduce preventive and therapeutic strategies in early stages, when they are likely to be more effective. At present, no method has been validated as a screening tool of atherosclerotic burden in both general population and patients with rheumatic diseases [131]. As a single marker may not be sufficient to determine the cardiovascular risk, a combination of different diagnostic investigations including biomarkers and imaging modalities can be utilised. In that respect, ADMA may be a valuable biochemical indicator of vascular disease and cardiopulmonary involvement as part of a multimarker risk tool. Results derived from large, hard endpoint trials are needed, to guide therapeutic targets in rheumatic diseases to minimize CVD co-morbidity, as well as provide insights into inflammatory mechanisms of atherosclerosis in general.

## Figures and Tables

**Table 1:** Overview of the studies exploring the clinical significance of ADMA in systemic inflammatory diseases.

Study	Patients	Parameters assessed	Assessment tools	ADMA	Associations
Surdacki *et al*. [87]	30 RA/20 controls	Atherosclerosis	Carotid U/S	↑RA	IMT, endothelial progenitor cells count
Turiel *et al*. [88]	25 RA/25 controls		Dipyridamole trans-thoracic stress U/S Carotid U/S	↑RA	CFR
Sandoo *et al*. [89]	67 RA/29 controls	Microvascular/macrovascular function Arterial stiffness	LDI with iontophoresis of ACh and SNP FMD with high-resolution U/S of the brachial artery Augmentation index	↑RA	No associations
Surdacki *et al*. [111]	20 RA	Autoimmunity	APCA		APCA
Kwaśny-Krochin *et al*. [103]	46 RA/50 controls	Disease activity	Inflammatory markers Disease activity and disability scores	↑RA	CRP, fibrinogen, DAS28, HAQ
Turiel *et al*. [104]	10 RA	Response to biologics		(−) after treatment	
Sandoo *et al*. [105]	35 RA				
Atzeni *et al*. [90]	22 PsA/35 controls	ED Atherosclerosis	Dipyridamole trans-thoracic stress U/S Carotid U/S	↑PsA	CFR
Sari *et al*. [91]	48 AS/38 controls	Disease activity CVD risk	Inflammatory markers	↑AS	CRP, cholesterol
Kemény-Beke *et al*. [92]	61 AS/26 OA	Disease activity	Inflammatory markers	↑AS	ESR, chest expansion
Kiani *et al*. [97]	200 SLE	Atherosclerosis Disease activity Autoimmunity	Coronary calcium score Inflammatory markers Autoantibodies		Coronary calcium, anti-dsDNA, ↓ complement, ESR
Perna *et al*. [98]	125 SLE	Atherosclerosis Arterial stiffness	Carotid U/S Augmentation index		Augmentation index
Bultink *et al*. [99]	107 SLE	CVD risk			↑ ADMA predicts CVD events
Dooley *et al*. [123]	45 SSc/19 PR/25 controls	ED	ADMA, nitration of proteins	↑SSc ↑dSSc	
Blaise *et al*. [126]	39 SSc/28 controls	Matrix remodelling ED	TIMP-1 ADMA	↑SSc	TIMP-1
Wipff *et al*. [127]	187 SSc/48 controls	Angiogenesis ED	Soluble endoglin ADMA	no difference	
Dimitroulas *et al*. [116]	66 SSc	SScPH	U/S	↑SScPH	
Dimitroulas *et al*. [117]	66 SSc/30 controls	SScPH	U/S	↑SScPH	6MWT
Dimitroulas *et al*. [118]	52 SSc/25 controls	Occult cardiac involvement	TDI	↑Cardiac disease	

ACh: acetylcholine; ADMA: asymmetric dimethylarginine; APCA: anticitrullinated protein antibodies; AS: ankylosing spondylitis; CFR: coronary flow reserve; CRP: *C*-reactive protein; CVD: cardiovascular disease; DAS28, disease activity score; dSSc: diffuse systemic sclerosis; ED: endothelial dysfunction; ESR: erythrocyte sedimentation rate; FMD: flow mediated dilatation; HAQ: health assessment questionnaire; IMT: intima-media thickness; LDI: laser Doppler imaging; PsA: psoriatic arthritis; PR: primary Raynaud’s; RA: rheumatoid arthritis; SLE: systemic lupus erythematosus; SSc: systemic sclerosis; SSc-PH: systemic sclerosis associated pulmonary hypertension; TDI: tissue Doppler imaging; TIMP-1: tissue inhibitor of matrix metalloproteinases-1; U/S: ultrasound; 6MWT: six minute-walking test.
